# Severe Acute Pancreatitis With Simultaneous Anterior ST-Elevation Myocardial Infarction in a 28-Year-Old: Navigating Conflicting Emergencies

**DOI:** 10.7759/cureus.114789

**Published:** 2026-08-19

**Authors:** Hind Alkazim, Fatin Naji, Ali AlNajjar, Mhmod Kadom, Mohamed Albreiki, Abdulaziz Albahri, Mohammed Abdul Muqsit Khan, Alya Alhammadi, Firas Yassin

**Affiliations:** 1 Cardiology, Dubai Health Authority, Dubai, ARE; 2 General Surgery, Rashid Hospital, Dubai, ARE; 3 Interventional Cardiology, Dubai Health Authority, Dubai, ARE

**Keywords:** acute pancreatitis, coronary thrombosis, oct (optical coherence tomography), primary percutaneous coronary intervention (pci), pseudoinfarction, st-segment-elevation acute coronary syndrome (ste-acs)

## Abstract

Severe acute pancreatitis is a systemic inflammatory disease that can lead to distant organ damage, including the myocardium. In rare cases, this can manifest as a myocardial infarction, posing significant diagnostic and therapeutic challenges. We report a case of acute pancreatitis with simultaneous anterior ST-elevation myocardial infarction in a 28-year-old man who presented with abdominal pain. Coronary angiography confirmed a thrombotic lesion in the left anterior descending artery. This case highlights the importance of considering true coronary pathology in patients with acute pancreatitis presenting with ECG changes. A review of the literature suggests that most reported cases occurred in males without conventional cardiovascular risk factors, implying alternative pathogenic mechanisms.

## Introduction

Acute pancreatitis is one of the most common gastrointestinal emergencies worldwide and the leading cause of hospitalization for gastrointestinal disease in the United States, accounting for approximately 200,000-275,000 admissions annually. It typically presents with acute-onset, severe epigastric pain and may range from a mild, self-limiting illness to severe disease complicated by multiorgan failure and systemic inflammatory response syndrome [1,2].

Although primarily an abdominal disorder, acute pancreatitis is well recognized to produce systemic cardiovascular manifestations. ECG abnormalities have been reported in more than half of affected patients, with ST-segment and T-wave changes occurring in up to one-quarter of cases. These abnormalities may closely resemble acute myocardial infarction despite the absence of obstructive coronary artery disease, a phenomenon commonly referred to as a “pseudo-infarction” pattern. Proposed mechanisms include electrolyte disturbances, autonomic imbalance, inflammatory cytokine release, coronary vasospasm, and direct myocardial injury. Consequently, distinguishing pancreatitis-related ECG changes from true acute coronary occlusion can be clinically challenging, particularly during the initial evaluation [3].

In contrast, the simultaneous occurrence of acute pancreatitis and true acute myocardial infarction is exceedingly rare, with only a limited number of cases reported in the literature. Although uncommon, this coexistence carries significant diagnostic and therapeutic implications because management priorities may conflict. Delayed recognition of coronary occlusion can result in irreversible myocardial injury, whereas inappropriate administration of antithrombotic therapy or urgent coronary intervention in patients with isolated pancreatitis may increase the risk of bleeding and other complications [3,4].

Acute coronary syndrome (ACS), encompassing ST-elevation myocardial infarction (STEMI), non-ST-elevation myocardial infarction, and unstable angina, remains a leading cause of morbidity and mortality worldwide. While ST-segment elevation on ECG is highly suggestive of acute coronary occlusion, its interpretation must always be integrated with the clinical presentation, cardiac biomarkers, imaging findings, and, when indicated, coronary angiography to differentiate true infarction from its mimics.

We report the case of a young adult presenting with severe acute pancreatitis and concurrent anterior STEMI confirmed by coronary angiography. This case highlights the diagnostic complexity of differentiating pseudo-infarction from true coronary thrombosis and emphasizes the importance of early multidisciplinary collaboration to balance timely reperfusion therapy with the competing therapeutic priorities imposed by severe pancreatitis.

## Case presentation

A previously healthy 28-year-old male presented to the emergency department (ED) with a three-day history of progressively worsening, severe generalized abdominal pain associated with mild sweating, nausea, vomiting, loss of appetite, decreased urination, and constipation. He denied fever, chest pain, shortness of breath, or recent trauma. Additionally, he denied previous episodes of similar symptoms. He denied past medical or surgical history. Family history was positive for diabetes mellitus in his mother, but was otherwise negative for cardiovascular conditions. He reported smoking one to two cigarettes per day for three years and denied alcohol consumption.

In the ED, he was hypertensive with a blood pressure of 141/105 mmHg and tachycardic with a pulse of 142 beats/minute. He was otherwise afebrile and maintained saturation on room air. On general inspection, the patient was alert and oriented to time, place, and person, but appeared to be in pain and was diaphoretic. The cardiovascular examination revealed tachycardia, but otherwise normal heart sounds, with no additional sounds or murmurs. Jugular venous pressure was normal. Auscultation of the lungs revealed no crackles or rales. Examination of the extremities showed no signs of peripheral edema. On abdominal examination, the abdomen was distended with generalized abdominal tenderness on palpation.

**Figure 1:**
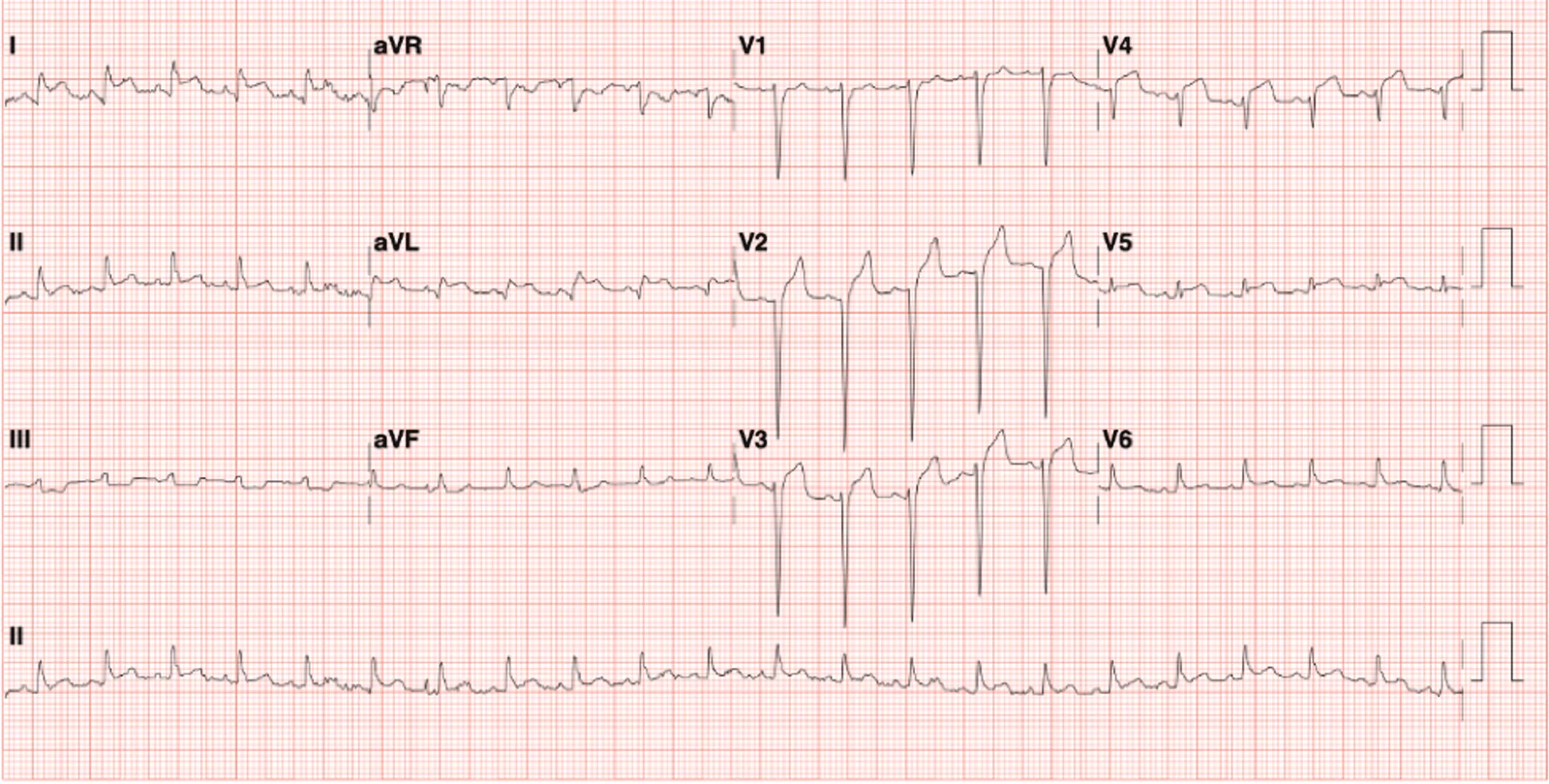
Patient’s ECG on admission showing sinus tachycardia with ST-segment elevation in the septal leads.

An ECG performed in the resuscitation bay revealed sinus tachycardia with ST-segment elevation in the anterior leads (Figure 1). Bedside echocardiography performed in the ED showed a normal ejection fraction (EF) with no regional wall motion abnormality or pericardial effusion. Laboratory investigations are shown in Table 1.

**Table 1:** Serial laboratory findings from admission through terminal deterioration. NA indicates that a result was not available for that time point. The early-course column combines the nearest available measurements during the early hospitalization; coagulation studies in this column reflect the closest available measurements. Serum bicarbonate and ABG bicarbonate are presented separately. BUN equivalents were calculated from the reported urea concentrations using BUN = urea/2.14 and rounded to the nearest whole number. EPO was sampled on admission. WBC: white blood cell count; CRP: C-reactive protein; PCT: procalcitonin; EPO: erythropoietin; PT: prothrombin time; INR: international normalized ratio; aPTT/PTT: activated partial thromboplastin time; BUN: blood urea nitrogen; ALP: alkaline phosphatase; ALT: alanine aminotransferase; CK-MB: creatine kinase-myocardial band; CPK: creatine phosphokinase; LDH: lactate dehydrogenase; HDL: high-density lipoprotein; LDL: low-density lipoprotein; HbA1c: hemoglobin A1c; ABG: arterial blood gas; FEU: fibrinogen-equivalent units

Laboratory test	Admission	Early course	ICU admission	Day of demise	Reference range/Note
WBC count	11.2 × 10⁹/L	13.3 × 10⁹/L	25.3 × 10⁹/L	6.2 × 10⁹/L	4.0–11.0 × 10⁹/L
Hemoglobin	23.0 g/dL	12.4 g/dL	11.4 g/dL	11.2 g/dL	13.8–17.2 g/dL
Hematocrit	68.6%	37.1%	34.9%	35.5%	40–50%
Platelet count	228 × 10³/µL	177 × 10³/µL	782 × 10³/µL	126 × 10³/µL	150–450 × 10³/µL
EPO	6.8 mIU/mL	NA	NA	NA	4.3–29.0 mIU/mL
D-dimer	NA	NA	NA	14.66 µg/mL FEU	<0.5 µg/mL FEU
PT	19.4 sec	16.6 sec	21.8 sec	25.6 sec	12.6–16.3 seconds
INR	1.38	1.23	1.57	1.06	0.8–1.1
aPTT/PTT	37.7 sec	43.2 sec	44.4 sec	61.3 sec	28–41 seconds
CRP	327 mg/L	299 mg/L	261 mg/L	315 mg/L	0–5 mg/L
PCT	NA	1.31 ng/mL	20.90 ng/mL	2.35 ng/mL	<0.05 ng/mL
Lactic acid	6.14 mmol/L	1.4 mmol/L	2.9 mmol/L	23 mmol/L	0.5–2.2 mmol/L
Serum bicarbonate	15 mmol/L	19.6 mmol/L	15.8 mmol/L	21.3 mmol/L	22–28 mmol/L
Creatinine	1.03 mg/dL	0.59 mg/dL	1.62 mg/dL	1.41 mg/dL	0.7–1.3 mg/dL
Urea/Calculated BUN	31 mg/dL (BUN ≈14 mg/dL)	81 mg/dL (BUN ≈38 mg/dL)	47 mg/dL (BUN ≈22 mg/dL)	66 mg/dL (BUN ≈31 mg/dL)	Urea: 12–40 mg/dL; BUN: 7–20 mg/dL
Total bilirubin	1.89 mg/dL	2.46 mg/dL	1.60 mg/dL	NA	0.1–1.2 mg/dL
ALP	115 IU/L	164 IU/L	101 IU/L	NA	44–121 IU/L
ALT	98 IU/L	57 IU/L	88 IU/L	NA	7–56 IU/L
Lipase	2,231 U/L	136 U/L	384 U/L	NA	10–140 U/L
Troponin T	1,770 ng/L	NA	53 ng/L	119 ng/L	<14 ng/L
CK-MB	44.1 ng/mL	NA	1.0 ng/mL	3.0 ng/mL	0–5 ng/mL
CPK	773 U/L	NA	NA	242 U/L	38–174 U/L
LDH	404 U/L	NA	NA	NA	105–222 U/L
pH	7.364	7.377	7.38	6.847	7.35–7.45
ABG bicarbonate	19.3 mmol/L	19.4 mmol/L	15.2 mmol/L	16 mmol/L	21–28 mmol/L
Sodium	130 mmol/L	131 mmol/L	133 mmol/L	151 mmol/L	136–145 mmol/L
Potassium	5.0 mmol/L	1.07 mmol/L	4.7 mmol/L	4.6 mmol/L	3.4–4.5 mmol/L
Total calcium	9.7 mg/dL	8.1 mg/dL	8.0 mg/dL	NA	9.0–10.2 mg/dL
Ionized calcium	1.12 mmol/L	1.07 mmol/L	1.11 mmol/L	1.11 mmol/L	1.15–1.29 mmol/L
Total cholesterol (fasting)	140 mg/dL	NA	NA	NA	<190 mg/dL
Triglycerides	124 mg/dL	NA	NA	NA	<150 mg/dL
HDL cholesterol	42 mg/dL	NA	NA	NA	>40 mg/dL
LDL cholesterol	73 mg/dL	NA	NA	NA	<115 mg/dL
Non-HDL cholesterol	98 mg/dL	NA	NA	NA	<145 mg/dL
Lipoprotein(a)	<20 nmol/L	NA	NA	NA	<75 nmol/L
HbA1c	5.3%	NA	NA	NA	<5.7%

While the patient had acute pancreatitis, given the clear ST elevation seen on ECG and elevated cardiac markers, the STEMI pathway was activated. He was administered 5 mg intravenous (IV) metoprolol as temporary management of tachycardia. Additionally, he was administered IV fentanyl for pain control and was loaded with 300 mg of aspirin. IV fluid maintenance therapy was started with careful monitoring of fluid balance for the treatment of acute pancreatitis. The patient was subsequently taken to the cardiac catheterization laboratory by the cardiology team. Imaging with CT of the abdomen was delayed in view of urgent percutaneous coronary intervention. In view of polycythemia, the hematology service was consulted, and they advised workup (erythropoietin, antiphospholipid, and myeloproliferative neoplasms, including Janus kinase 2 levels, anti-beta-2 glycoprotein antibody, anti-cardiolipin antibody, and lupus anticoagulant), which came back negative, and phlebotomy once feasible.

**Figure 2:**
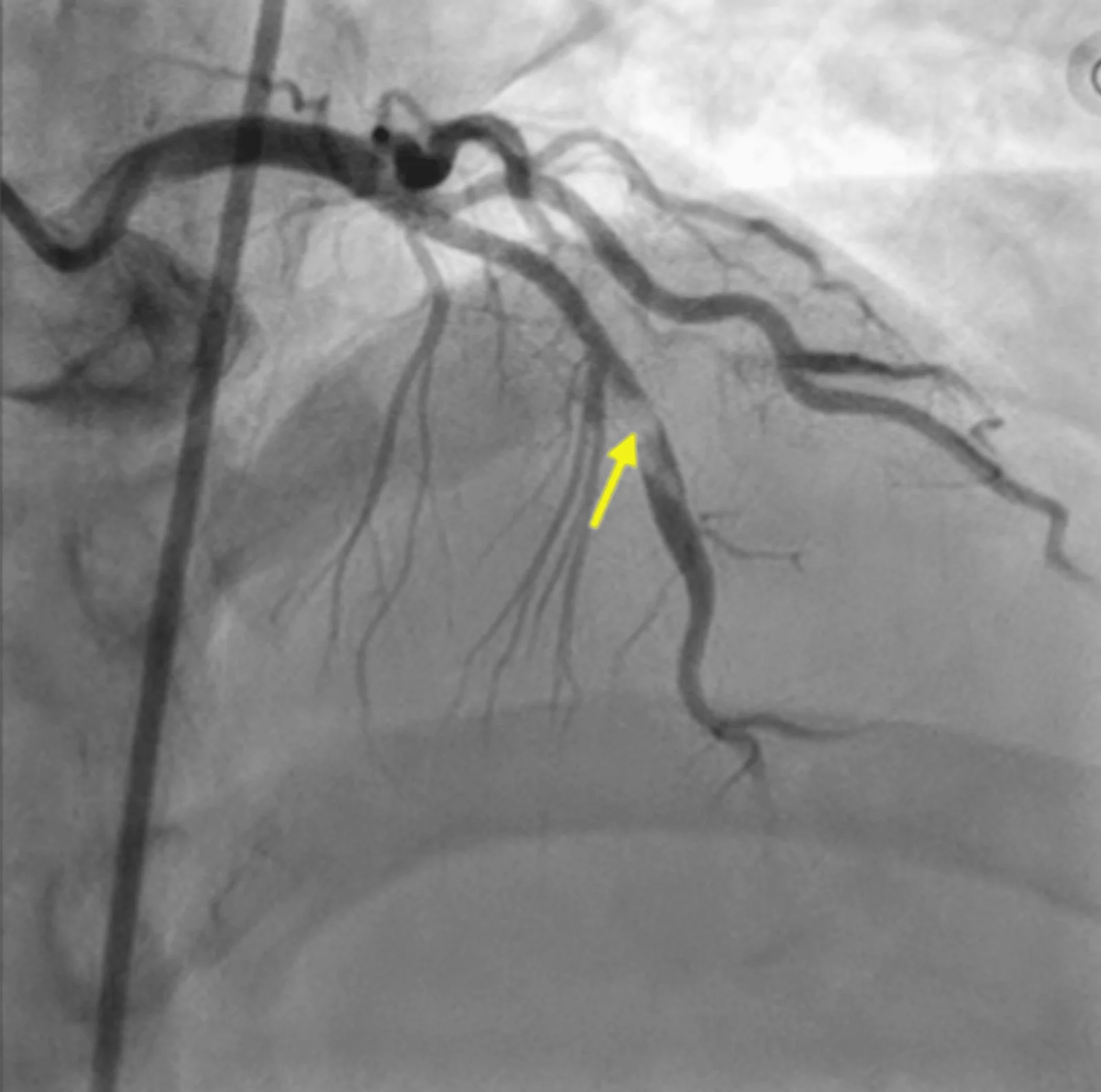
First coronary angiogram revealing critical 90-99% stenosis of the mid-LAD with the presence of a heavy intramural thrombus (yellow arrow). LAD: left anterior descending artery

**Figure 3:**
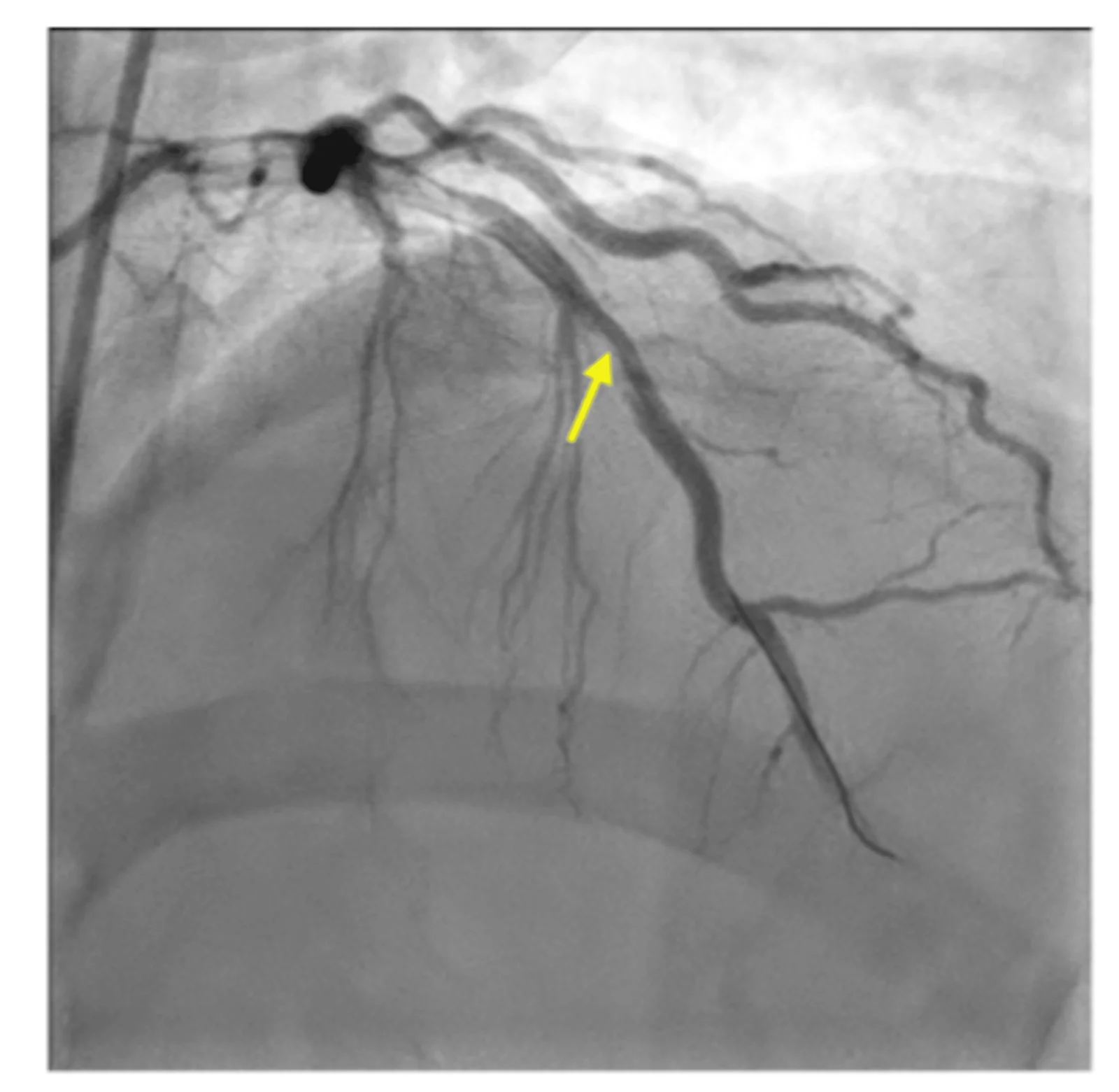
Restoration of TIMI Flow III after successful angioplasty and thrombectomy of mid-LAD lesion (yellow arrow). TIMI: Thrombolysis in Myocardial Infarction; LAD: left anterior descending artery

In the cardiac catheterization laboratory, the coronary arteries were accessed through the right femoral artery. Coronary angiography revealed a normal left main artery, normal left circumflex artery, a normal and dominant right coronary artery, and a thrombotic lesion in the mid-left anterior descending artery (LAD) causing 90-99% occlusion of the artery (Figure 2). The LAD was engaged using a Judkins left catheter, which led to severe spasm of the LAD with no reflow. This was treated with the administration of intracoronary nitrates and adenosine, which restored the flow. A balloon was then used to dilate the mid-LAD, and the thrombectomy of the identified thrombus was performed, following which Thrombolysis in Myocardial Infarction (TIMI) III flow was achieved (Figure 3). Due to the initial suspicion of the patient having an underlying thrombotic disease, a stent was not placed due to the high risk of stent thrombosis. The procedure was completed, and the patient was scheduled for a relook angiography. An infusion of 12.5 mg of tirofiban was initiated for 48 hours, along with ticagrelor (180 mg loading dose followed by 90 mg twice daily).

**Figure 4:**
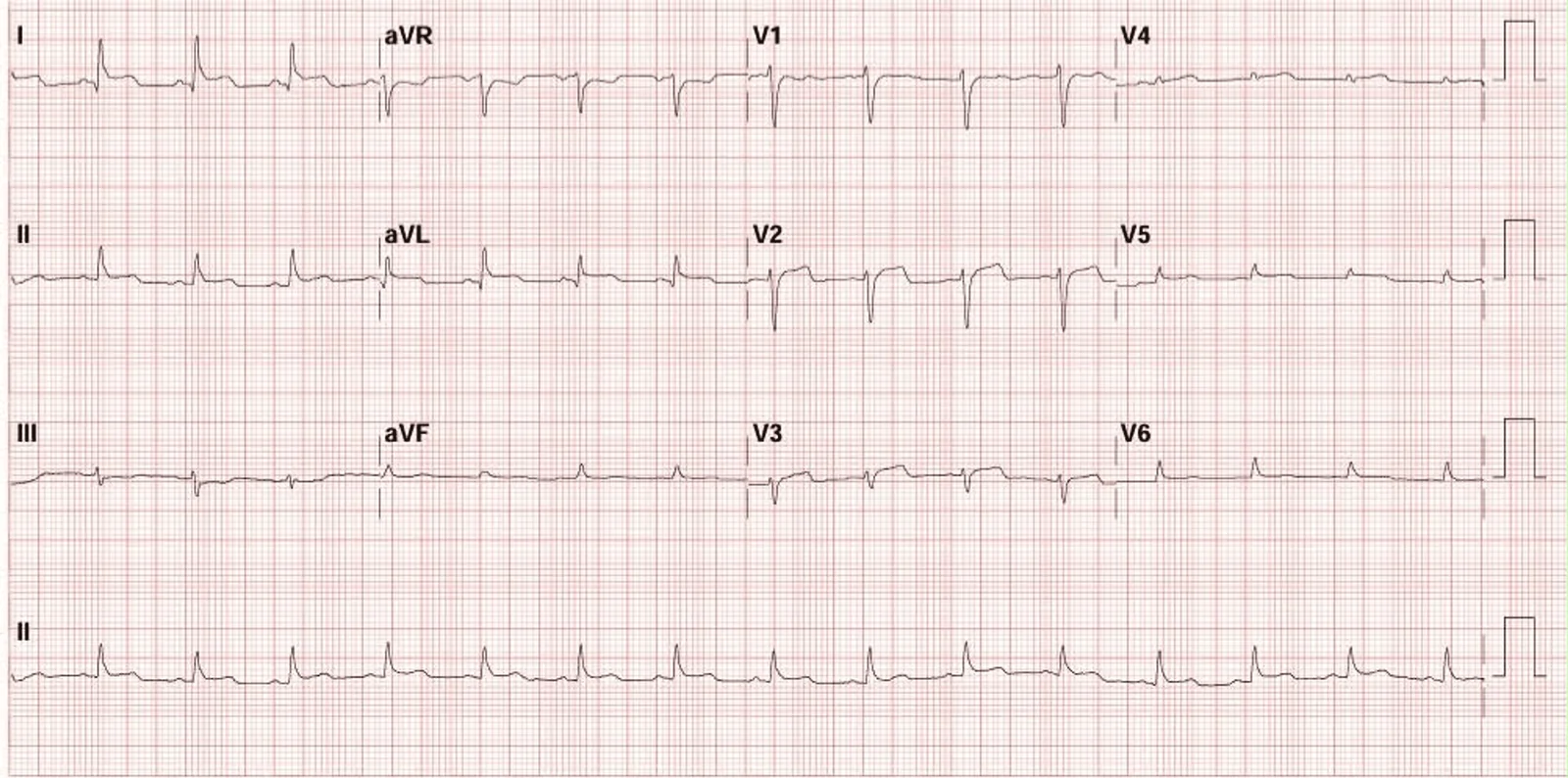
Post-coronary angiography and angioplasty ECG showing ST-T elevation resolution with biphasic T waves in V2-V3.

**Figure 5:**
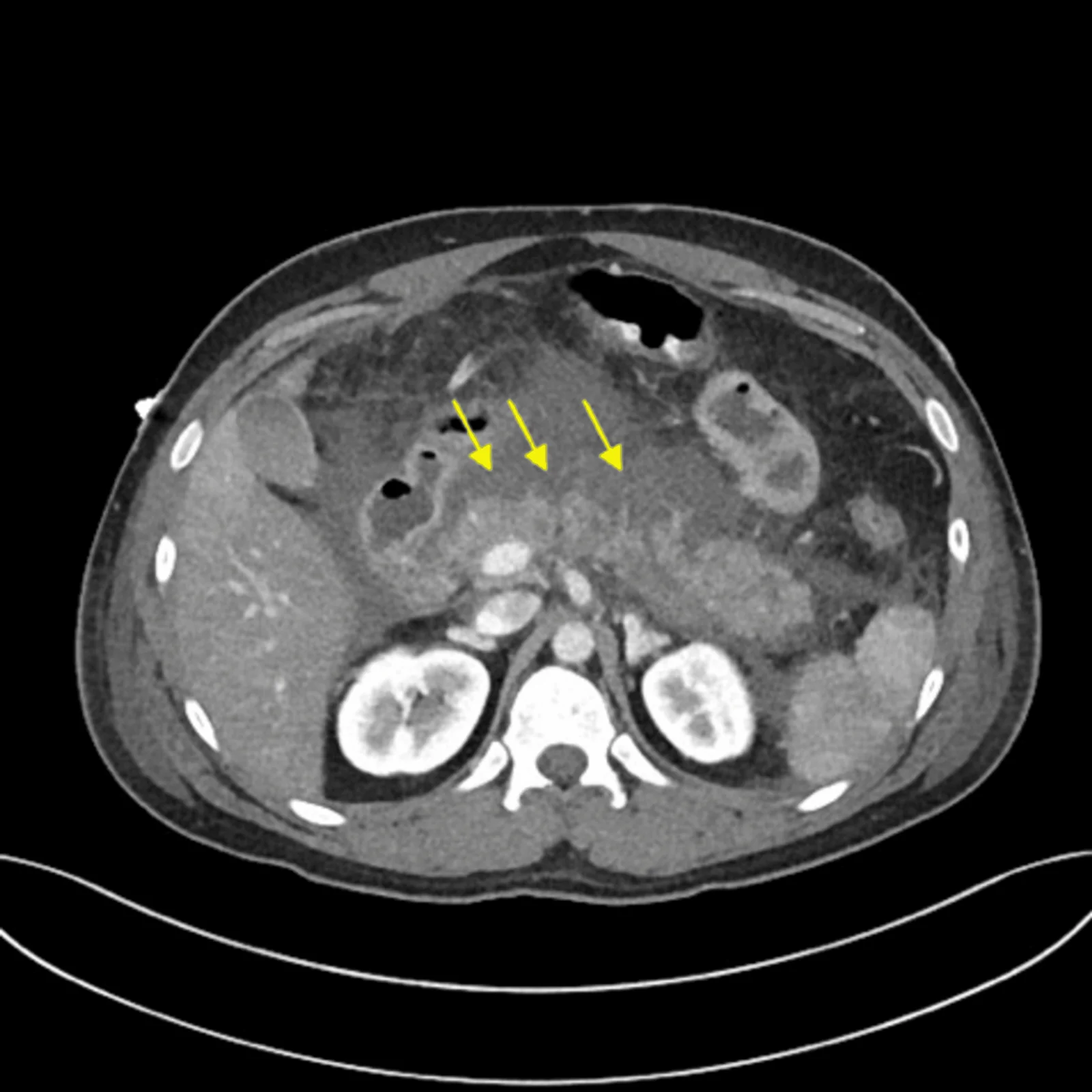
Axial section of a CT scan of the abdomen and pelvis revealing peripancreatic edema (yellow arrows).

Following angiography, the patient was admitted to the cardiology ward and was closely followed by the surgical team. A post-angiography ECG was obtained and showed ST-T-segment elevation resolution (Figure 4). An ultrasound scan of the abdomen done on day one of admission showed a mildly enlarged liver, minimal bilateral pleural effusions, mild perinephric fluid, and ascites. No calculi were observed in the gallbladder or common bile duct (CBD). Departmental echocardiography was also performed, which revealed an EF of 50-55%, normal heart chamber sizes, and no valvular abnormalities. The patient was started on 1 g IV meropenem three times daily in view of high inflammatory markers. A CT scan of the abdomen and pelvis with contrast (Figure 5), performed on day three due to rising inflammatory markers, revealed a swollen pancreas with significant edema in the peripancreatic and retroperitoneal fat, confirming the diagnosis of acute pancreatitis. Over the next few days, he reported improvement in abdominal pain and bloating. The previously noted abdominal distension and tenderness reduced. Additionally, mild impairments in the patient’s kidney function improved with co-management by the medical team. Subsequent ECGs showed sinus tachycardia with mild residual ST elevation in leads I, AVL, and V1 to V4.

Despite clinical improvement, the patient had persistently deranged liver function tests. Although total hepatitis A virus (HAV) antibodies were positive, HAV immunoglobulin M (IgM) levels were negative, ruling out acute viral hepatitis. Due to these findings and the uncertainty of possible biliary pancreatitis, the patient underwent magnetic resonance cholangiopancreatography (MRCP) on day 11 of admission, which showed no dilation of the intrahepatic or common bile ducts, as well as no evidence of calculi in the gallbladder or CBD.

**Figure 6:**
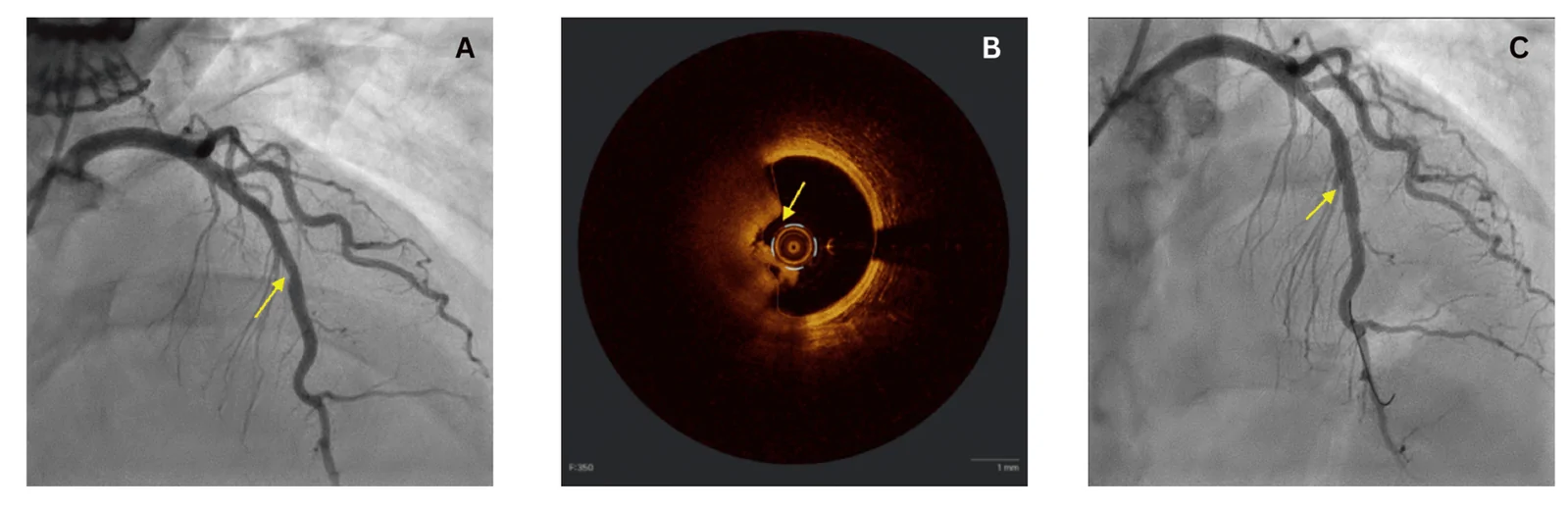
(A) Relook angiogram revealing <50% stenosis of LAD involving the origin of first diagonal with normal anterograde flow. (B) Intraoperative OCT confirming the presence of ulcerated plaque in LAD. (C) Successful angioplasty of LAD and placement of one drug-eluting stent. LAD: left anterior descending artery; OCT: optical coherence tomography

The patient underwent relook angiography on day 13 of admission, which revealed a non-flow-limiting (<50%) stenosis of the mid-LAD (Figure 6A) involving the origin of the first diagonal artery. There was additionally an acute occlusion of the distal LAD. The clot seen in the previous angiography procedure had completely resolved. Optical coherence tomography (OCT) confirmed an ulcerated plaque (Figure 6B), which was stented (Figure 6C). OCT confirmed good expansion, apposition, and no edge dissection. The patient was then counseled to take aspirin for life and ticagrelor for 12 months.

**Figure 7:**
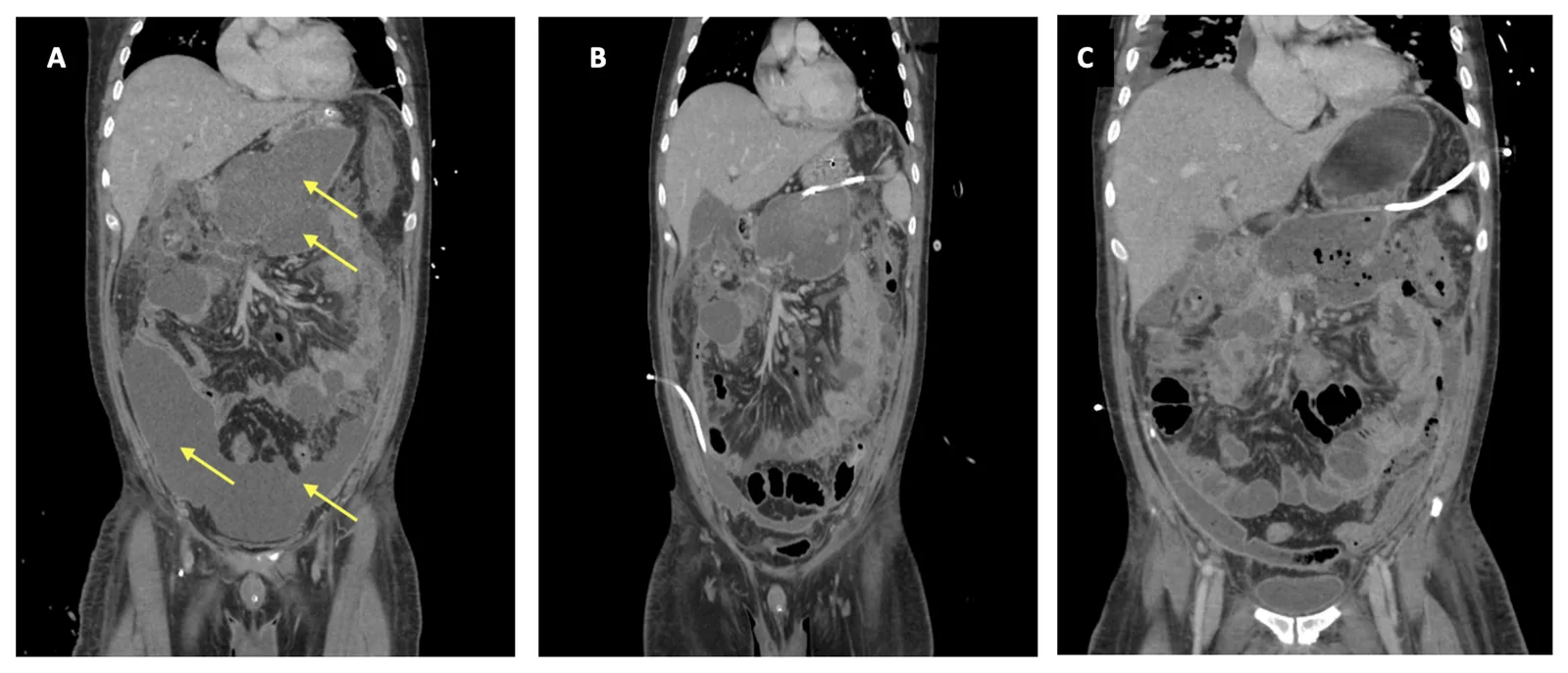
(A) CT scan of the abdomen and pelvis with contrast revealing large intraperitoneal walled off collections. (B) CT scan of the abdomen and pelvis showing two 12-French drains placed in perigastric and lower abdominal collections. (C) CT scan of the abdomen and pelvis showing walled off collections with reactive wall thickening and slightly increased air densities within the old collection which is likely due to a communication fistula between collection and adjacent transverse colon.

Following the second coronary intervention, the patient’s cardiac status remained stable; however, his pancreatitis progressed despite intensive medical management. He developed extensive necrotizing pancreatitis with multiple pancreatic and peripancreatic collections, necessitating transfer to the surgical intensive care unit. Broad-spectrum antimicrobial therapy was continued, and CT-guided bilateral percutaneous drainage catheters were inserted after imaging demonstrated infected walled-off pancreatic necrosis. Drain fluid cultures subsequently grew *Escherichia coli*, confirming infected pancreatic necrosis. Despite repeated drain manipulation and replacement, the patient remained febrile with persistently elevated inflammatory markers and progressive enlargement of the necrotic collections (Figures 7A, 7B). His clinical course was further complicated by bilateral pleural effusions and worsening respiratory failure requiring thoracentesis followed by endotracheal intubation. Repeat CT demonstrated enlarging necrotic collections with evidence of fistulous communication between the pancreatic necrotic cavity and the transverse colon (Figure 7C). Following repositioning of the abdominal drains, feculent output was noted, prompting emergency exploratory laparotomy.

Intraoperatively, feculent peritonitis, extensive pancreatic necrosis with a large pancreatic abscess cavity, and two fistulous communications between the transverse colon and the necrotic cavity were identified. Necrosectomy, extensive abdominal washout, primary repair of the colonic fistulas, bilateral chest tube insertion, and temporary abdominal closure using a Bogota bag were performed because severe intraoperative hemodynamic instability precluded definitive abdominal closure.

Despite maximal postoperative intensive care, including mechanical ventilation, vasopressor support, broad-spectrum antimicrobial therapy, and ongoing resuscitation, the patient developed refractory septic shock with progressive multiorgan failure. Shortly after surgery, he experienced profound circulatory collapse followed by brady-asystolic cardiac arrest. Advanced Cardiovascular Life Support was performed without return of spontaneous circulation, and he died on hospital day 42 and day 28 of intensive care from refractory septic shock secondary to infected necrotizing pancreatitis complicated by feculent peritonitis and pancreatic-colonic fistulas. Table 2 presents a timeline of the patient’s clinical course.

**Table 2:** Timeline of the patient’s hospital course. TIMI: Thrombolysis in Myocardial Infarction; LAD: left anterior descending artery; MRCP: magnetic resonance cholangiopancreatography; CBD: common bile duct; OCT: optical coherence tomography; ICU: intensive care unit

Hospital day	Clinical course
Day 1	Presentation with severe abdominal pain and vomiting. ECG showed anterior ST-segment elevation. Coronary angiography demonstrated a 90-99% thrombotic mid-LAD occlusion. Balloon angioplasty and thrombectomy were performed, restoring TIMI III flow. No stent was placed initially
Day 3	CT of the abdomen and pelvis demonstrated acute pancreatitis with pancreatic swelling and significant peripancreatic and retroperitoneal edema
Day 11	MRCP showed no intrahepatic or common bile duct dilatation and no gallbladder or CBD calculi
Day 13	Relook coronary angiography showed resolution of the initial LAD thrombus. OCT demonstrated an ulcerated mid-LAD plaque, which was stented. Later that day, the patient developed hypotension, fever, worsening inflammatory markers, and septic shock, requiring ICU admission and vasopressor support
Day 17	Repeat CT demonstrated progression of acute pancreatitis with large retroperitoneal and intraperitoneal loculated, partially walled-off collections. Respiratory status deteriorated, requiring NIV
Day 18	Electively intubated because of increasing ventilatory requirements and inability to tolerate NIV weaning
Day 20	CT-guided drainage of intra-abdominal collections was performed, with two 12-French drainage catheters placed
Day 22	Clinical improvement following drainage; successfully extubated after progressive ventilatory weaning
Days 23-41	Continued complicated course of severe necrotizing pancreatitis with persistent/recurrent intra-abdominal infection and sepsis despite drainage and intensive supportive management
Day 42	Underwent emergency exploratory laparotomy, pancreatic necrosectomy and abdominal washout, primary closure of two transverse-colon fistulas, bilateral chest-tube placement, and temporary abdominal closure with a Bogota bag
Subsequent course	Postoperatively developed refractory septic shock and multiorgan failure, followed by bradycardic/asystolic cardiac arrest. Resuscitation was unsuccessful
Outcome	Demise

## Discussion

Mechanisms linking acute pancreatitis and myocardial infarction

Acute pancreatitis is associated with profound systemic inflammation and marked intravascular volume depletion, both of which contribute to a transient prothrombotic state. In patients with severe acute pancreatitis, extensive third-spacing of fluids due to increased capillary permeability results in significant plasma volume loss and hemoconcentration, often reflected by markedly elevated hemoglobin and hematocrit. Unlike true polycythemia, this represents relative polycythemia, in which the red blood cell mass remains unchanged while plasma volume is substantially reduced [5].

Hemoconcentration has important rheological consequences. The resulting increase in blood viscosity slows coronary blood flow, impairs microvascular perfusion, promotes platelet margination and activation, and increases the likelihood of thrombus formation. Experimental and clinical studies have demonstrated that elevated hematocrit increases shear stress and enhances platelet adhesion to the vascular endothelium, thereby predisposing to arterial thrombosis. In acute pancreatitis, admission hematocrit has also been recognized as a marker of disease severity and is associated with pancreatic necrosis and systemic complications, suggesting that severe hemoconcentration reflects an intense inflammatory and hypovolemic state [5,6].

Simultaneously, acute pancreatitis induces a systemic inflammatory response characterized by the release of pro-inflammatory cytokines, including tumor necrosis factor-α and interleukin-6, which activate endothelial cells, platelets, and the coagulation cascade while suppressing endogenous fibrinolysis. The combination of endothelial dysfunction, platelet activation, increased thrombin generation, and impaired fibrinolysis creates a transient hypercoagulable state that further increases the risk of arterial thrombosis [6].

In the present case, these mechanisms likely acted synergistically. The patient’s hemoglobin of 23 g/dL and hematocrit of 68.6% on presentation, followed by normalization and subsequent anemia after aggressive IV fluid resuscitation, strongly support severe hemoconcentration secondary to dehydration rather than primary polycythemia. This transient hyperviscous state, superimposed on the inflammatory and hypercoagulable milieu of severe acute pancreatitis, provides a biologically plausible mechanism for coronary thrombus formation and angiographically confirmed STEMI. The dramatic correction of hemoglobin and hematocrit following volume replacement further supports relative polycythemia as the principal mechanism linking acute pancreatitis to coronary thrombosis in this patient.

Although additional mechanisms, including autonomic dysfunction, coronary vasospasm, direct myocardial toxicity from circulating pancreatic enzymes, and electrolyte disturbances, may contribute to myocardial injury in acute pancreatitis, these processes are more commonly implicated in transient ischemic ECG changes or pseudo-infarction syndromes. In contrast, the presence of angiographically confirmed coronary occlusion in our patient suggests that dehydration-induced hemoconcentration, together with inflammation-driven hypercoagulability, was the predominant pathophysiological pathway leading to acute coronary thrombosis [6,7].

Interpretation of cardiac biomarkers in acute pancreatitis

Elevations in cardiac biomarkers are frequently observed in patients with acute pancreatitis, even in the absence of obstructive coronary artery disease, complicating differentiation from ACS [8].

Cardiac troponin elevation has been repeatedly documented but does not reliably indicate acute myocardial infarction. A prospective time-course study demonstrated persistent elevation of cardiac troponin T during early hospitalization despite the absence of ischemic ECG changes or clinical evidence of coronary thrombosis. Similarly, creatine kinase-myocardial band (CK-MB) levels may be modestly elevated because of inflammatory myocardial injury rather than true myocardial necrosis. However, higher CK-MB concentrations have also been associated with severe acute pancreatitis and the development of persistent organ failure [8].

Natriuretic peptides, particularly N-terminal pro b-type natriuretic peptide, are frequently elevated in acute pancreatitis and are believed to reflect myocardial strain secondary to inflammatory stress, hemodynamic instability, and volume shifts rather than primary heart failure.

ECG abnormalities further complicate diagnosis, as acute pancreatitis may produce pseudo-infarction patterns including ST-segment elevation, T-wave inversion, and QTc prolongation that closely resemble acute myocardial infarction. These abnormalities are often transient and occur without obstructive coronary disease, reflecting inflammatory myocardial injury and autonomic imbalance. Consequently, isolated elevations in cardiac biomarkers or ECG abnormalities should not automatically be interpreted as evidence of ACS [8].

Accurate interpretation of cardiac biomarkers requires integration of the clinical presentation, serial biomarker measurements, ECG findings, echocardiographic assessment, and, when clinically indicated, coronary angiography [8-10].

In the present case, the patient presented with marked anterior ST-segment elevation, elevated cardiac biomarkers, and a pronounced systemic inflammatory response. Initial bedside echocardiography demonstrated preserved left ventricular systolic function without regional wall motion abnormalities, findings that could initially suggest a pseudo-infarction pattern. However, given the potentially catastrophic consequences of delayed reperfusion in true STEMI, coronary angiography remained essential. Angiography demonstrated thrombotic occlusion of the mid-LAD with restoration of TIMI III flow following thrombectomy, confirming true STEMI. Subsequent OCT identified an ulcerated culprit plaque requiring stent implantation, highlighting the value of intracoronary imaging in guiding definitive management.

Role of imaging in differentiating pseudo-infarction from acute coronary syndrome

Cross-sectional abdominal imaging further contributed to the diagnostic evaluation. Contrast-enhanced CT confirmed severe acute pancreatitis with extensive retroperitoneal inflammatory changes, an extracardiac process capable of producing ECG abnormalities and a systemic inflammatory response [6,7]. Previous reports have similarly demonstrated that abdominal imaging may identify pancreatitis as the primary diagnosis in patients presenting with pseudo-infarction patterns, particularly when abdominal symptoms predominate [7]. Nevertheless, abdominal imaging should not delay emergent coronary angiography when STEMI is suspected.

Diagnostic challenges and differential diagnosis

This case illustrates several important diagnostic challenges. The predominance of abdominal pain together with the absence of traditional cardiovascular risk factors may bias clinicians toward non-cardiac diagnoses, potentially delaying reperfusion therapy [7]. Conversely, aggressive ACS-directed therapy, including dual antiplatelet therapy, anticoagulation, and invasive coronary angiography, may increase bleeding risk and complicate management in severe acute pancreatitis [6,7].

The patient’s young age together with a profound inflammatory and hypercoagulable state may also have contributed to the significant coronary thrombotic burden, consistent with previous reports suggesting that systemic inflammation may precipitate plaque instability and coronary thrombosis [5-7].

The differential diagnosis includes pseudo-infarction secondary to acute pancreatitis, concomitant acute pancreatitis with true ACS, Takotsubo cardiomyopathy, myocarditis, coronary vasospasm, and hypercoagulable or embolic coronary disease [7-9].

Management dilemma and multidisciplinary decision-making

Management in such cases requires close multidisciplinary collaboration because competing therapeutic priorities frequently exist [6,7]. Aggressive IV fluid resuscitation, the cornerstone of acute pancreatitis management, may conflict with the hemodynamic goals following myocardial infarction, particularly in the presence of left ventricular dysfunction. Decisions regarding antithrombotic therapy, stent implantation, and the timing of repeat angiography in our patient benefited from coordinated input from cardiology, gastroenterology, hepatobiliary surgery, hematology, and intensive care teams.

The decision to defer stent implantation during the index procedure because of concerns regarding thrombosis within a highly inflammatory and hypercoagulable environment illustrates the importance of individualized procedural planning rather than rigid adherence to standardized ACS algorithms [7,11,12].

## Conclusions

Taken together, this case underscores that acute pancreatitis and true STEMI are not mutually exclusive and may coexist with significant clinical consequences. Imaging, both cardiac and non-cardiac, plays a pivotal role in differentiating pseudo-infarction patterns from ACS, guiding therapeutic sequencing, and mitigating diagnostic delay. Given the rarity of simultaneous presentation and the management conflicts that arise, early multidisciplinary engagement is essential to optimize outcomes.

## References

[1] Banks PA, Freeman ML (2006). Practice guidelines in acute pancreatitis. Am J Gastroenterol.

[2] Pezzilli R, Barakat B, Billi P, Bertaccini B (1999). Electrocardiographic abnormalities in acute pancreatitis. Eur J Emerg Med.

[3] Jain A, Singh A, Shah K, Grossman SA (2025). Acute Coronary Syndrome. Updated.

[4] Vasantha Kumar A, Mohan Reddy G, Anirudh Kumar A (2013). Acute pancreatitis complicated by acute myocardial infarction - a rare association. Indian Heart J.

[5] Li L, Tan Q, Wu X (2024). Coagulopathy and acute pancreatitis: pathophysiology and clinical treatment. Front Immunol.

[6] Luo Y, Li Z, Ge P (2021). Comprehensive mechanism, novel markers and multidisciplinary treatment of severe acute pancreatitis-associated cardiac injury - a narrative review. J Inflamm Res.

[7] Khan U, Petrechko O, Sagheer S, Majeed H, Zaidi SH, Wasty N, Sheikh AB (2023). Acute pancreatitis and myocardial infarction: a narrative review. Cardiology.

[8] Barassi A, Pezzilli R, Romanelli MC, Banfi G, Merlini G, d'Eril GM (2015). Serum markers of myocardial damage in acute pancreatitis: a prospective time course study. Pancreas.

[9] Zhao B, Sun S, Wang Y (2021). Cardiac indicator CK-MB might be a predictive marker for severity and organ failure development of acute pancreatitis. Ann Transl Med.

[10] Xiao H, Huang JH, Zhang XW (2017). Identification of potential diagnostic biomarkers of acute pancreatitis by serum metabolomic profiles. Pancreatology.

[11] Pan L, Niu Z, Ren S, Zhang L, Pei H, Zhang Z, Gao Y (2025). Cardiac complications in acute pancreatitis: an under-diagnosed clinical concern. World J Emerg Med.

[12] Yokoi M, Ito T, Yamamoto J (2023). Intraprocedural thrombotic events during percutaneous coronary intervention due to acquired antithrombin deficiency-related heparin resistance successfully treated with antithrombin gamma supplementation. Intern Med.

